# ^18^F-PSMA-1007 PET/CT Performance on Risk Stratification Discrimination and Distant Metastases Prediction in Newly Diagnosed Prostate Cancer

**DOI:** 10.3389/fonc.2021.759053

**Published:** 2021-10-28

**Authors:** Zhuonan Wang, Anqi Zheng, Yunxuan Li, Weixuan Dong, Xiang Liu, Wang Yuan, Fan Gao, Xiaoyi Duan

**Affiliations:** ^1^ PET/CT Unit, Department of Medical Imaging, The First Affiliated Hospital of Xi’an Jiaotong University, Xi’an, China; ^2^ Clinical Research Center, The First Affiliated Hospital of Xi’an Jiaotong University, Xi’an, China

**Keywords:** primary prostate cancer, ^18^F-PSMA-1007 PET/CT, risk stratification, distant metastasis, tPSA, SUVmax

## Abstract

**Objective:**

To evaluate the prediction performance of ^18^F-PSMA-1007 PET/CT and clinicopathologic characteristics on prostate cancer (PCa) risk stratification and distant metastatic prediction.

**Materials and Methods:**

A retrospective analysis was performed on 101 consecutively patients with biopsy or radical prostatectomy proved PCa who underwent ^18^F-PSMA-1007 PET/CT. The semi-quantitative analysis provided minimum, maximum and mean standardized uptake (SUVmin, SUVmax and SUVmean) of PCa. Association between clinicopathologic characteristics (total prostate-specific antigen, tPSA and Gleason Score, GS) and PET/CT indexes were analyzed. The diagnostic performance of distant metastatic on PET/CT parameters, tPSA and GS was evaluated using logistic regression analyses. A path analysis was conducted to evaluate the mediating effect of tPSA level on the relation between semi-quantitative parameters of primary tumors and metastatic lesions.

**Results:**

The PET/CT parameters were all higher in high risk stratification subgroups (tPSA>20 ng/mL, GS ≥ 8, and tPSA>20 ng/mL and/or GS ≥ 8, respectively) with high sensitivity (86.89%, 90.16% and 83.61%, respectively). The SUVmax, tPSA and GS could effectively predict distant metastatic with high sensitivity of SUVmax (90.50%) compared with tPSA (57.14%) and GS (55.61%). With a cutoff value of 29.01ng/mL for tPSA, the detection rate of distant metastasis between low and high prediction tPSA group had statistical differences (50.00% vs. 76.60%, respectively; P = 0.006) which was not found on guideline tPSA level (P>0.05). 6/15 (40%) patients tPSA between 20ng/mL to 29.01ng/mL without distant metastases may change the risk stratification. Finally, tPSA had a partial mediating effect on SUVmax of primary tumors and metastases lesions.

**Conclusion:**

The ^18^F-PSMA-1007 PET/CT SUVmax has a higher sensitivity and can be an “imaging biomarker” for primary PCa risk stratification. The prediction tPSA level (29.01 ng/mL) is more conducive to the assessment of distant metastasis and avoid unnecessary biopsy.

## Introduction

Prostate cancer (PCa) is the highest malignant male tumor and one of the leading causes of mortality among men worldwide (1, 2). The biological behaviors of PCa malignancy are largely heterogeneous, directly impacting prognostic grouping, and treatment options (3). In addition, assessments of the distant metastatic status for PCa patients have recently received increasing attention due to the heightening mortality rate (4, 5). Therefore, the precise systemic staging of primary PCa risk stratification before treatment plays a crucial role in designing the management strategy for the individualized treatment option. According to both American Urological Association (AUA) and the European Association of Urology (EAU) guidelines, patients with total prostate-specific antigen (tPSA) > 20 ng/mL and/or Gleason Score ≥ 8 are high-risk, the probability of distant metastasis and mortality will increase significantly and may not suitable for active surveillance programs, radical prostatectomy or radiotherapy treatment (6–8). However, tPSA is organ-specific but not tumor-specific, the biological behaviors of prostate malignancy are largely heterogeneous, and the specificity of the ability of tPSA to reflect distant metastasis remains debatable (6). Using tPSA as the only indicator for risk stratification discrimination and distant metastases prediction may causing in large numbers of unnecessary prostate biopsies (9–12). Also, elderly patients with severe comorbidities or undergoing anticoagulation therapy may not be the optimal candidates for biopsies and may cause adverse effects and higher costs (10). In these cases, it is urgent to find objective and accurate imaging biomarker for risk stratification classification with noninvasive approach based on imaging analysis.

The prostate-specific membrane antigen (PSMA) is a type II transmembrane glycoprotein that is primary expressed in prostatic tissues, and its expression correlated with the degree of malignancy and further increases in metastatic (13, 14). The ability of PSMA to easily penetrate tissues and diffuse with solid tumor lesions can reflect the statuses of metastasis (3, 15, 16). Prior studies show that PSMA PET/CT is superior to conventional imaging methods for lymph node metastatic detection, and that the pre-treatment tPSA level and Gleason Score are associated with the PSMA uptake in primary PCa (17, 18). Furthermore, the Maximum Standardized Uptake Value (SUVmax) is the most commonly used semi-quantitative parameter in PET/CT and prior studies has already used to assess the degree of malignancy of PCa and predict extended pelvic lymph node metastases in intermediate to high-risk PCa patients by ^68^Ga-PSMA-11 or ^68^Ga-PSMA-617 (19). ^18^F-PSMA-1007 is advantaged by its higher spatial resolution images and non-urinary excretion that reduces urinary clearance, this approach bears a great potential to facilitate the detection of primary PCa and metastatic lesions (20, 21). However, to our knowledge, no prior studies have employed ^18^F-PSMA-1007 PET/CT to evaluate the diagnostic performance in risk stratification and distant metastases prediction in primary PCa.

The present study aims to retrospective investigated the role of ^18^F-PSMA-1007 PET/CT semi-quantitative parameters correlation among newly diagnosed PCa imaging, tPSA levels and Gleason Score, and to evaluate the prediction performance of ^18^F-PSMA-1007 PET/CT and clinicopathologic characteristics on PCa risk stratification and distant metastatic prediction.

## Materials and Methods

### Patients

**Figure 1:**
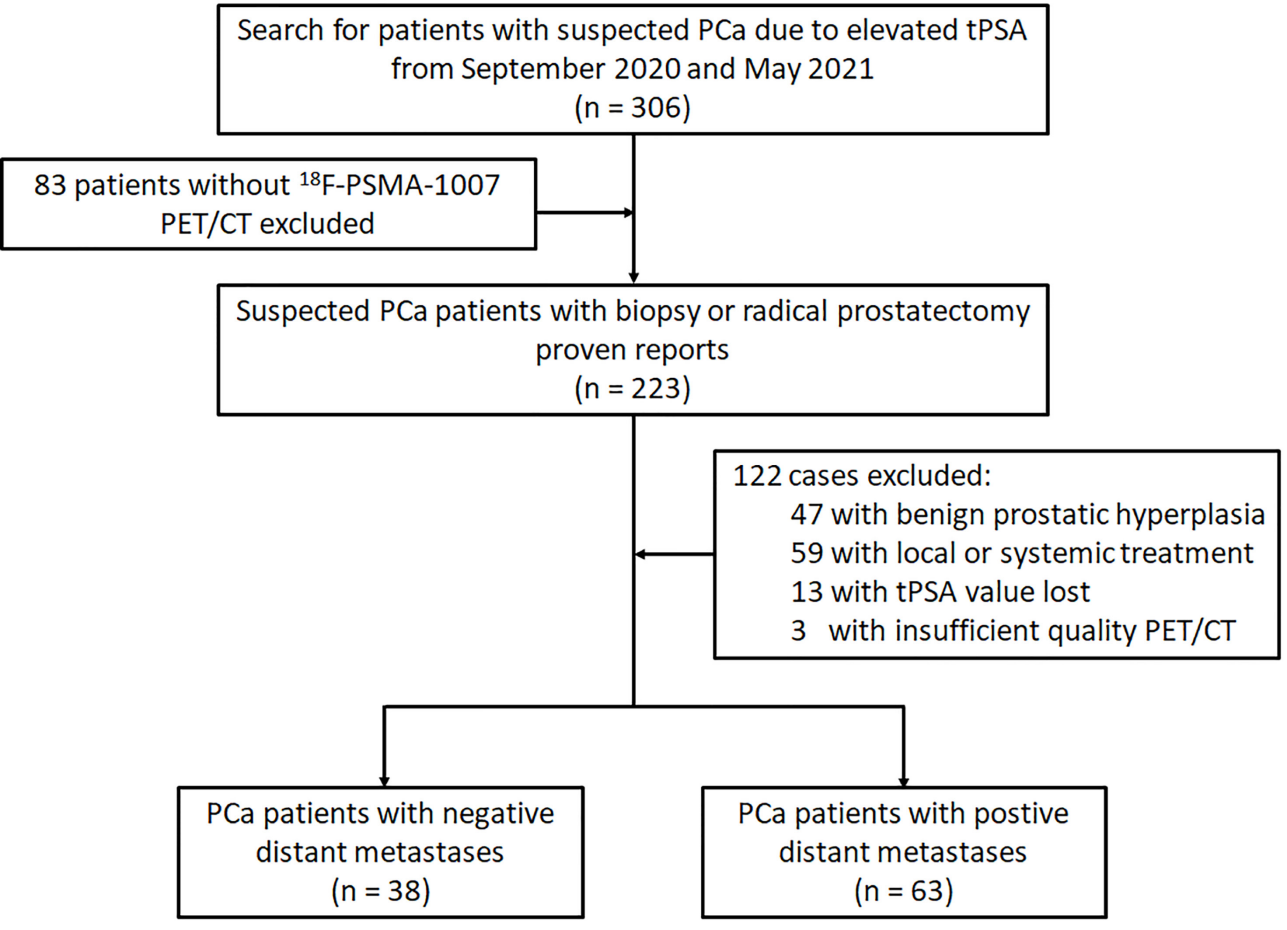
Flowchart of the PCa patient’s cohort.

The study has been approved by the institutional review board (No. 2019LSYZD-J1-H) and was conducted in accordance with the Declaration of Helsinki. All subjects signed an informed consent form. We performed retrospective analysis for 101 patients with primary PCa confirmed by biopsy or radical prostatectomy between September 2020 and May 2021. All participants included in the data analysis were evaluated by ^18^F-PSMA-1007 PET/CT and had tPSA value measured within 4 weeks prior to the ^18^F-PSMA-1007 PET/CT imaging. Diagnosis of PCa proven through histological examination served as reference for the PET imaging analyses (18). Patients were excluded from analysis if they 1) had received local or systemic treatment, 2) lacked histological examination proven diagnosis or tPSA value, 3) had incomplete imaging data. The flowchart of patient enrollment is provided in **Figure 1**.

### ^18^F-PSMA-1007 Acquisition and Imaging Analysis

All ^18^F-PSMA-1007 PET/CT data was acquired on a PET/CT scanner (Gemini 64TF, Philips, Netherlands) at a single location. Radiolabeling was performed using a fully automated radiopharmaceutical synthesis device based on a modular concept (MINItrace, GE Healthcare, USA). Over 99% radiochemical purification yield ^18^F-PSMA was obtained and examined by both radio-thin layer chromatography (TLC) and high-performance liquid chromatography (HPLC) analysis. Patients received intravenous injection of ^18^F-PSMA-1007 (3.7 MBq/kg body weight), and completed PET and CT scans 90 minutes after the injection. Low-dose CT scans from head to the proximal thighs (pitch 0.8 mm, 60 mA, 140 kV [peak], tube single turn rotation time 1.0 s and 5-mm slice thickness) for PET attenuation were acquired (pitch 0.8 mm, automatic mA, 140 kV [peak] and 512 × 512 matrix). Whole-body PET scans were performed in three-dimensional mode (emission time: 90 s per bed position, scanned at a total of 7-10 beds).

**Figure 2:**
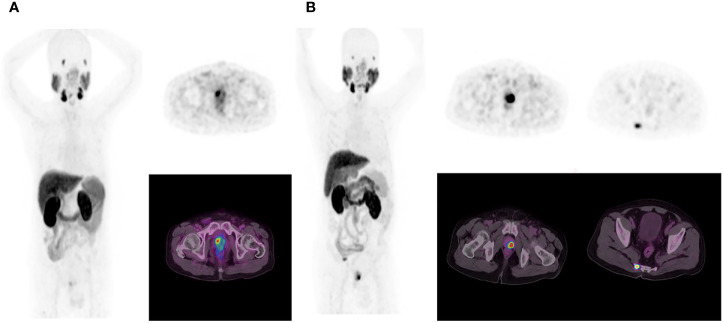
In the first patient **(A)**, ^18^F-PSMA-1007 PET/CT results show primary prostate cancer on whole-body maximum intensity projection (MIP) image (left). Axial PET image (right up) and axial fused image (right bottom) show primary tumor in the right prostate cancer lobe. In the second patient **(B)**, ^18^F-PSMA-1007 PET/CT results show primary prostate cancer and tailbone metastasis on MIP image (left).

All ^18^F-PSMA-1007 PET/CT images were analyzed using Fusion Viewer software in the Extended Brilliance Workstation (EBW, Philips, Netherlands). Two experienced nuclear medicine specialists jointly interpreted all ^18^F-PSMA-1007 PET/CT scans, and performed comprehensive analysis of available clinical data. Consensuses were achieved through discussion when conclusions between the two specialists were discordant. The PET indexes (including SUVmin, SUVmax and SUVmean) of the primary PCa was calculated automatically with a manually adapted isocontour threshold centered on lesions with focally increased uptake corresponding to the tumor site verified by TRUS biopsy or radical prostatectomy (17). The PET indexes values were also calculated for metastases lesions. The positive lesion was defined by an uptake higher than the local background and not associated with physiologic uptake per the guideline of the Society of Nuclear Medicine and Molecular Imaging and the European Association of Nuclear Medicine (22, 23). The identified metastases were also consistent with PCa lesions pathologic tracer accumulation (6, 22, 23). The PET/CT distant metastasis positive lesions were also composite validated by other imaging approaches (bone scan and MR) as a reference, and the patients were followed for tPSA measurements, imaging follow-up (PET/CT, bone scan and MR), disease management as the metastatic definition reference (24, 25). PET/CT scan findings was classified as (a) primary tumor, and (b) distance metastasis (abdominal lymph nodes, bone and internal organs) (**Figure 2**).

### Statistical Analysis

Descriptive statistics was used to display patient data as median, mean, standard deviation range or percentages, where applicable. Correlation among PET/CT indexes (including SUVmin, SUVmax and SUVmean) and variables were evaluated with Spearman’s rank correlation coefficient. Wilcoxon- Mann‐Whitney U test was used to test the subgroups (tPSA > 20 ng/mL vs tPSA ≤ 20 ng/mL; Gleason Score ≥ 8 vs Gleason Score<8; High-risk vs Low-Intermediate risk) PET indexes differences. The risk stratification discrimination of PET/CT indexes and distant metastases status prediction combined PET/CT indexes with clinicopathologic characteristics were all assessed using receiver operating characteristic (ROC) curve analysis. The PET/CT parameters and clinicopathologic characteristics were used to construct logistic regression prediction model of metastasis findings on ^18^F-PSMA-1007. The metastasis diagnostic efficiency of the model was evaluated by ROC curve and the best threshold of tPSA and PET/CT indexes performance was based on Youden index. Statistical significance of the association between positive/negative metastasis findings on ^18^F-PSMA-1007 and clinicopathologic characteristics was assessed with chi-squared test. The path analyses to examine the potential mediating role of tPSA level on PET/CT index across different metastatic statuses. A significance level of α = 0.05 (two-tailed) was applied. Statistical analyses were performed using IBM SPSS Statistics version 13.0, GraphPad Prism software, version 8.4 and MedCalc version 19.0.

## Results

Demographic information and clinical characteristics of the participants were summarized in **Table 1**. A total of 208 lesions were identified using ^18^F-PSMA-1007 PET/CT, including 101 primary prostate tumors and 107 distant metastases lesions. The median SUVmax of all primary PCa lesions was 26.00 (range: 5.95-101.89), which was significantly higher than that of the metastasis’s lesions (median SUVmax: 16.90, range: 5.44-150.24). Comparing the subgroup with tPSA > 20 ng/mL and that with tPSA ≤ 20 ng/mL, Gleason Score ≥ 8 and that with Gleason Score < 8, and high risk and that with low-intermediate risk, the PET/CT semi-quantitative parameters (SUVmin, SUVmax and SUVmean) of the first group were all higher than that of the second group in **Table 2**. The PET/CT indexes were all significantly correlated with the tPSA (r_s_ = 0.405, 0.380, 0.418, respectively, *P < 0.001*), Gleason Score (r_s_ = 0.407, 0.339, 0.387, respectively, *P <.001*) and risk stratification (r_s_ = 0.432, 0.354, 0.430, respectively, *P <.001*).

**Table 1:** Demographic and clinical characteristics of the 101 study participants.

Characteristic	Value
**Age (years)**	
Median (range)	72 (50-90)
Mean ± SD	71.39 ± 8.72
**tPSA (ng/mL)**	
Median (range)	24.97 (0.17-2139)
Mean ± SD	123.57 ± 296.50
**Non-metastatic Patients (%)**	38 (37.6%)
**Metastatic Patients (%)**	63 (62.4%)
**Gleason Score**	3 (3.0%)
6	35 (34.7%)
7	21 (20.8%)
8	42 (41.5%)
9	
**Risk Group**	
Low-Intermediate risk	38 (37.6%)
High risk	63 (62.4%)
**Number and percentage of metastatic malignant lesions**	
Extrapelvic Lymph node metastases	67/107 (62.6%)
Bone metastases	40/107 (37.4%)

**Table 2:** ^18^F-PSMA-1007 PET/CT parameters of different tPSA, Gleason Score and risk stratification subgroups.

Categorical variable	PSA ≤ 20 (n = 40)	PSA > 20 (n = 61)	Sig	GS < 8 (n =38)	GS ≥ 8 (n = 63)	Sig	Low-Intermediate risk (n = 22)	High-risk(n = 79)	Sig
SUVmin									
Mean ± SD (range)	6.85 ± 5.84 (1.41-23.8)	10.68 ± 7.27 (1.89-37.95)	*P = 0.01*	6.77 ± 5.53 (1.41-21.18)	10.61 ± 7.37 (1.75-37.95)	*P = 0.01*	4.92 ± 4.77 (1.41-21.12)	32.37 ± 19.77 (1.75-37.95)	*P = 0.002*
SUVmax									
Mean ± SD (range)	22.35 ± 16.30 (5.95-57.83)	33.84 ± 20.23 (7.06-101.89)	*P = 0.006*	22.80 ± 14.99 (5.95-57.83)	33.21 ± 20.96 (7.06-101.89)	*P = 0.013*	18.22 ± 14.13 (5.95–57.83)	15.88 ± 10.49 (7.06-101.89)	*P = 0.004*
SUVmean									
Mean ± SD (range)	10.75 ± 8.80 (2.67-36.19)	16.42 ± 10.77 (3.72-57.85)	*P = 0.01*	10.69 ± 8.15 (2.67-31.75)	16.27 ± 11.04 (3.72-57.85)	*P = 0.012*	8.05 ± 7.28 (2.67-31.75)	10.34 ± 7.04 (3.72-57.85)	*P = 0.003*

**Figure 3:**
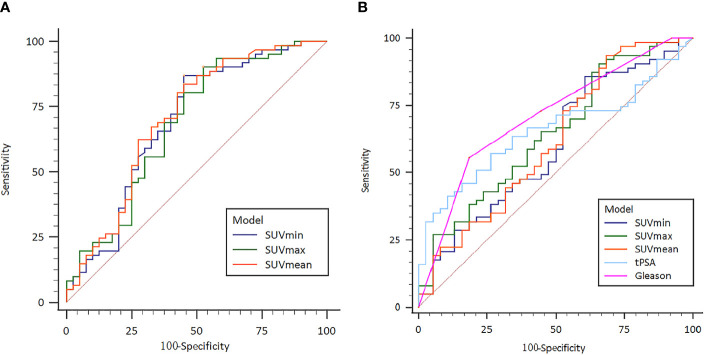
**(A)** Receiver operating characteristic (ROC) curve of the ^18^F-PSMA-1007 PET/CT indexes for prostate cancer risk stratification; **(B)** Receiver operating characteristic (ROC) curve of the ^18^F- PSMA-1007 PET/CT prostate cancer metastasis risk prediction.

To be specific, risk stratification model for PCa was constructed based on semi-quantitative parameters. Area under the ROC curve (AUC) was analyzed with 0.692 (95% CI: 0.593 - 0.780) with sensitivity 86.89% and specificity 55.00% for the SUVmin model (Cut-off value 6.56), 0.684 (95% CI: 0.584 - 0.773) with 90.16% and 47.50% for the SUVmax model (Cut-off value 31.19) and 0.706 (95% CI: 0.607 - 0.792) with 83.61% and 55.00% for the SUVmean model (Cut-off value 10.22) (**Figure 3A**). This study also tests the potential value of ^18^F-PSMA-1007 PET/CT in predicting the risk of PCa metastasis. The AUC, sensitivity and specificity of SUVmin, SUVmax, SUVmean, tPSA and Gleason Score were measured respectively. The AUC results were 0.602 (95% CI: 0.500 - 0.698) with sensitivity 85.71% and specificity 39.47% for the SUVmin model (Cut-off value 3.35, P >.05); AUC 0.645 (95% CI: 0.543 - 0.738) with sensitivity 90.50% and specificity 34.22% for the SUVmax model (Cut-off value 13.76, P <.05), AUC 0.619 (95% CI: 0.517 - 0.714) with sensitivity 93.71% and specificity 31.63% for the SUVmean model (Cut-off value 13.76, P <.05), AUC 0.656 (95% CI: 0.555-0.748) sensitivity with 57.14% and specificity 73.68% for the tPSA model (Cut-off value 29.01, P <.05) and AUC 0.709 (95% CI: 0.610 - 0.795) with sensitivity 55.61% and specificity 81.64% for the Gleason Score model (Cut-off value 8, P <.05) (**Figure 3B**).

To further evaluate the diagnostic strength of PET/CT indexes (SUVmax and SUVmean), tPSA level and Gleason Score for metastasis, SUVmax, SUVmean, tPSA (tPSA low: ≤20 ng/mL and tPSA high: > 20 ng/mL) and Gleason Score (low-intermediate: <8 and high: ≥ 8) were entered as independent variables in a logistics regression. The metastasis status was entered as a binary outcome variable. As shown in **Table 3**, the logistic regression found the SUVmax, tPSA level (high or low) and Gleason Score (low-intermediate risk or high-risk) were independent predictors for metastasis status. We also applied multivariate logistic regression for metastasis diagnosis. The SUVmax and Gleason Score (low-intermediate risk or high-risk) were stable for metastasis status prediction (OR 1.081, P = .040; OR 2.602, P = .042, respectively). The tPSA level (high or low) had no significant difference in multivariate logistic regression results. Details can be found in **Supplementary Materials**.

**Table 3:** Logistic analyses of factors predicting prostate cancer metastasis.

Categorical variable	OR	95% CI	P
**SUVmax**	1.032	1.005-1.059	*0.018*
**Gleason Score** **(high vs low-intermediate)**	3.120	1.179-8.257	*0.019*
**SUVmean**	1.040	0.994-1.088	0.087
**tPSA (high vs low)**	2.389	1.043-5.472	*0.038*

tPSA, total PSA; OR, odds ratio; CI, confidence interval. SUVmax and SUVmean here represent the PET/CT indexes of the primary tumor.

**Figure 4:**
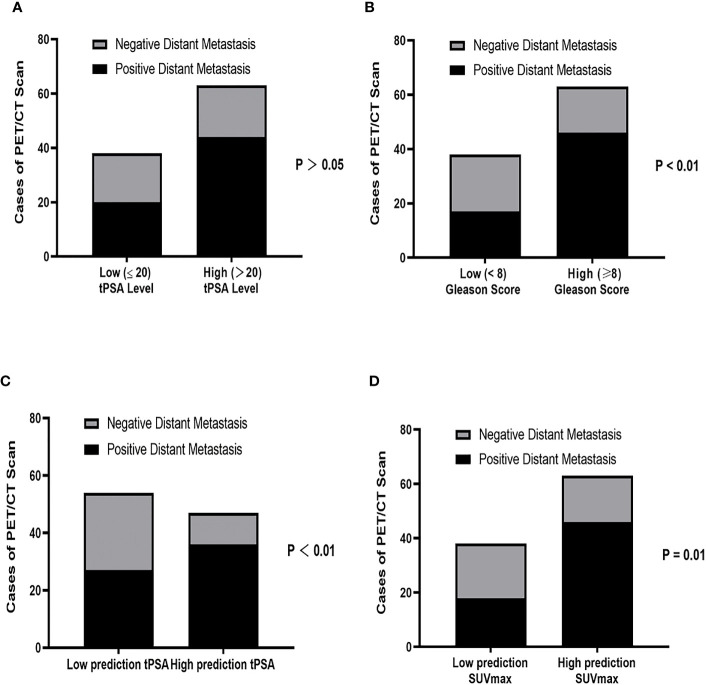
Correlation between **(A)** tPSA level (tPSA high: >20 ng/mL vs tPSA low: ≤20 ng/mL), **(B)** Gleason Score (low-intermediate: <8 vs high: ≥8), **(C)** Prediction tPSA level (tPSA<29.01 ng/mL vs tPSA ≥ 29.01 ng/mL) and **(D)** Prediction SUVmax (SUVmax ≤ 13.76 vs SUVmax>13.76) for positive distant metastasis findings on 18F PSMA-1007 PET/CT. tPSA, total PSA; PCa, prostate cancer.

We further analyzed the correlation between tPSA level (tPSA low and tPSA high), Gleason Score (low-intermediate and high), SUVmax and positive distant metastasis findings on ^18^F-PSMA-1007 PET/CT. The detection rate of distant metastasis was lower in the low-PSA compared with that in the high PSA group with no significance difference (52.63% vs. 69.84%, respectively; P >.05; **Figure 4A**). The detection rate of distant metastasis was lower in the low-intermediate Gleason Score group than in the high Gleason Score group (44.73% vs. 73.02%, respectively; P = .004; **Figure 4B**). We next sought to determine the tPSA level for optimal predicting the metastasis findings based on prior ROC curve. Basing on the cutoff value of 29.01 ng/mL for the prediction tPSA level, we categorized patients into a low prediction tPSA (tPSA ≤ 29.01 ng/mL) group and a high prediction tPSA group (tPSA>29.01 ng/mL). The detection rate of distant metastasis was lower in the low prediction tPSA compared with that in the high prediction tPSA group with statistical significance difference (50.00% vs. 76.60%, respectively; P = .006; **Figure 4C**). When applied the optimal prediction value of SUVmax (13.76), the high prediction SUVmax had higher detection rate for distant metastasis (47.39% vs.73.02%, respectively; P <.05; **Figure 4D**). In order to better prove the benefit from predicting PSA levels for risk stratification, we retrospectively analyzed primary PCa patients with tPSA between 20ng/mL to 29.01ng/mL. The ^18^F-PSMA-1007 PET/CT results of PCa patients found that 6 out of 15 (40%) did not have distant metastases.

**Figure 5:**
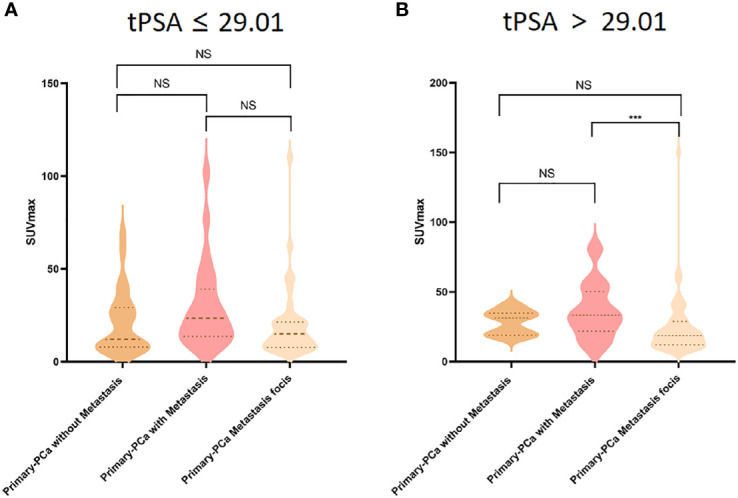
Comparison of ^18^F-PSMA-1007 SUVmax uptake in primary prostate tissue without metastasis (dark yellow violin box), primary prostate tissue with metastasis (pink violin box) and primary prostate cancer metastasis tissues (light yellow violin box) in tPSA ≤ 29.01 **(A)** and tPSA>29.01 **(B)** tPSA, total PSA; PCa, prostate cancer. ***P < 0.05, NS, No statistical difference.

To better identify the association between prediction tPSA value and SUVmax, we divided PCa patients into six subgroups based on their tPSA level (i.e., tPSA ≤ 29.01 ng/mL, tPSA>29.01 ng/mL) and metastasis staging (i.e., primary prostate tissue without metastasis, primary prostate tissue with metastasis, and primary PCa metastasis focis). ANOVA analysis found that SUVmax was significantly different among the high prediction tPSA subgroups (P = .001) but not within the low prediction tPSA subgroups (P >.05). Post-hoc analysis showed that, among the high prediction tPSA subgroups, only the primary prostate tissue with metastasis group and the primary PCa metastasis foci group demonstrated significantly different SUVmax level (P <.001) (**Figure 5**).

**Figure 6:**
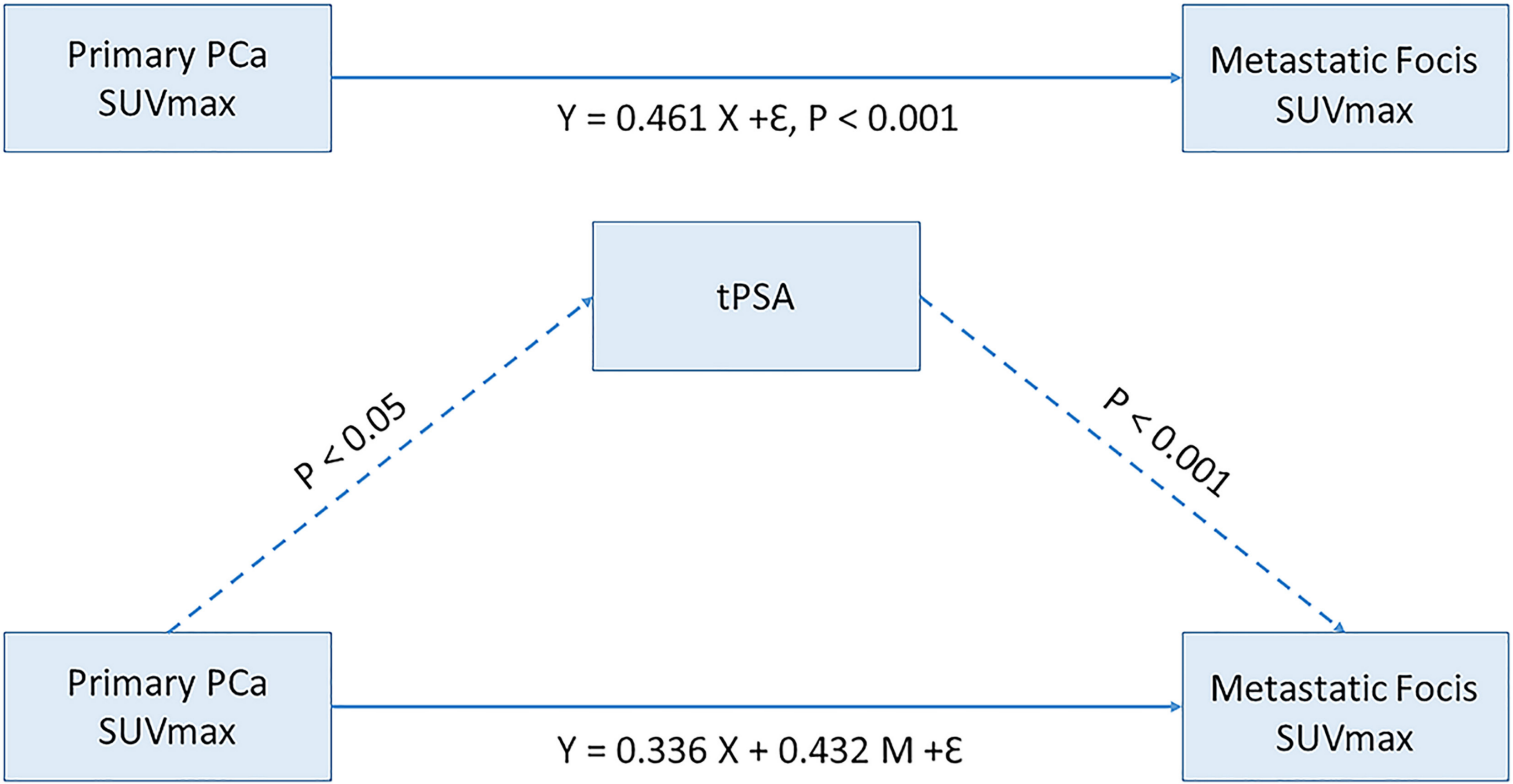
Mediating effect model between primary prostate cancer SUVmax and Metastatic focis. tPSA, total PSA; PCa, prostate cancer.

To evaluate the mediation role of tPSA level on SUVmax, we constructed a path analysis model by including SUVmax of primary PCa lesions as the predictor, tPSA level as the mediator, and SUVmax of metastasis foci as the outcome. A positive correlation was found between the primary PCa SUVmax and metastasis foci SUVmax (P <.001). tPSA was found to have a partial mediating effect between the SUVmax values of primary tumors and metastases lesions (P < .05) (**Figure 6**).

## Discussion

The present study analyzed the application value of ^18^F-PSMA-1007 PET/CT to detect risk stratification discrimination and distant metastases prediction in primary PCa. The SUVmax can be used as an “imaging biomarker” for distant metastasis risk prediction in primary PCa. Higher tPSA levels may be more likely to benefit from PET/CT detection of distant metastases. The SUVmax can effectively identify PCa heterogeneity of the primary tumor and metastasis lesions. Reference to the prediction tPSA level (29.01 ng/mL) can at least be a partial but important contribution to risk stratification and avoiding unnecessary invasive examinations. The tPSA has a partial mediating effect between the primary tumor and metastases lesions.

PSMA overexpression in primary PCa was correlated with advanced tumor malignant status with higher tPSA level and Gleason Score (15). Prior studies have shown the SUVmax of ^68^Ga-PSMA PET/CT associated with tPSA and Gleason Score (3, 17, 26). This study investigated the use of semi-quantitative parameters to determine the risk stratification, similarly correlation was also found between ^18^F-PSMA -1007 PET/CT parameters with both tPSA and Gleason Score. For PCa risk stratification, the SUVmin, SUVmax and SUVmean can accurately identify high tPSA level (tPSA > 20), high Gleason Score (≥ 8) and high-risk (tPSA > 20 or/and Gleason Score ≥ 8) primary PCa patients The ROC curve analysis indicated that these three semi-quantitative parameters have high sensitivity (86.89%, 90.16% and 83.61%, respectively) to screen out all PCa patients for high risk prediction and may satisficed clinical needs.

PCa metastasis risk prediction included both intra-pelvic and distant metastases, however, the treatment options of different location metastases may be quite different (7, 8). Series studies have demonstrated that PSMA PET/CT is more efficient than traditional imaging methods for detecting distant metastases in primary PCa patients and reflect the malignancy and staging PCa, preventing patients from undergoing repeated inspections and invasive biopsy (27–32). Our study explored the prediction value of the distant metastatic risk of primary PCa with ^18^F-PSMA -1007 PET/CT. The distant metastatic risk prediction model constructed by SUVmax, tPSA and Gleason Score can be used as independent factors of distant metastatic assessment. In the process of visual assessment, there is heterogeneity and false positive rate of lesions in distant location outside the prostate. When pathological results are not available, it may affect the choice of treatment. According to our results, SUVmax with an optimal value 13.76 has a higher sensitivity 90.50% compared with both tPSA (57.14%) and Gleason Score (55.61%), the positive detection rate will increase significantly, which may provide a reference for the diagnosis of distant metastasis. We also analyzed the correlation between distant metastases status and high-risk factors (tPSA, Gleason Score and SUVmax). Similar with previous results, the detection rate of distant metastasis increases with primary tumor malignancy and the tPSA level (33, 34). ^18^F-PSMA -1007 PET/CT positive distant metastasis PCa patients had a higher Gleason Score and SUVmax with statistical difference compared with PET/CT negative patients.

Prior studies using a tPSA cutoff value of 30 ng/ml in groups of men showed diagnose performance for primary PCa ranging from 90% to 95.7% (10). Among those who were not diagnosed with malignant PCa through biopsy, bone scan still detected positive metastases. The current study further found that the detection rate of distant metastasis was lower in the low prediction tPSA compared with that in the high prediction tPSA group, and higher detection rate compared with former tPSA (73.68% vs 69.84%). Furthermore, patients with tPSA between 20ng/mL to 29.01ng/mL, the ^18^F-PSMA-1007 PET/CT results found that 6 out of 15 (40%) did not have distant metastases. Based on the original guidelines may lead to unnecessary biopsy in some patients and the prediction tPSA level an at least be a partial but important contribution to risk stratification and avoiding unnecessary biopsy.

To understand how tPSA level may help differentiate the primary tumor from the distant metastasis, we grouped the PCa patients based on their prediction tPSA level. The SUVmax difference between primary tumors and metastatic lesions in metastatic PCa patients with a high prediction tPSA level (> 29.01ng/mL). Primary PCa can be a heterogeneous multifocal tumor, and may lead to metastatic lesions with varying characteristics (35–40). This finding may further reflect the specificity of the source of distant metastases and prostate characteristics at different tPSA levels. Considering the potential value of tPSA and SUVmax and the differential performance of these two indicators in the primary tumors and metastatic lesions, we recommended that when the patient’s tPSA is higher than the prediction level, the patients might be benefit from ^18^F-PSMA -1007 PET/CT scan for distant metastasis detection.

Our study was limited by the retrospective data collection and a relatively small sample size. Future work in this area should consider combining PET/CT imaging findings with pathological results of distant metastatic lesions to improve overall diagnostic accuracy. In addition, benign hyperplasia and inflammation may interfere with the SUVmax measurements of PCa. Methods to improve the accuracy of SUVmax measurement may help increase detection specificity. We also plan to implement the analytic approach for sites of metastases in larger datasets currently being collected in the lab with the hope of the association between sites of metastases and tPSA level.

In summary, ^18^F-PSMA-1007 PET/CT is of good application value in the risk stratification and distant metastatic of primary PCa. When tPSA is higher than the prediction level, the probability of distant metastasis will increase. The newly tPSA prediction level may change the risk stratification and patient’s treatment options.

## Data Availability Statement

The data that support the findings of this study are available from the corresponding author upon reasonable request.

## Ethics Statement

The studies involving human participants were reviewed and approved by the institutional review board of The First Affiliated Hospital of Xi’an Jiaotong University. The patients/participants provided their written informed consent to participate in this study.

## Author Contributions

ZW draft the manuscript, contributed to the conception and design, analysis and interpretation of data. AZ contributed to the design and analysis of data. YL contributed to the analysis and interpretation of data. WD contributed to the conception and design, and acquisition of data. XL contributed to the conception and design, and acquisition of data. WY contributed to the acquisition of data and analysis of data. FG contributed to the conception and design, analysis and interpretation of data. XD contributed to revise of the manuscript critically for important intellectual content. All authors contributed to the article and approved the submitted version.

## Funding

This study was supported by the 2020 Natural Science Basic Research Program of Shaanxi Province, China (2020JZ-38) and 2020 Clinical Research Project of The First Affiliated Hospital of Xi’an Jiaotong University (XJTU1AF-CRF-2020-008).

## Conflict of Interest

The authors declare that the research was conducted in the absence of any commercial or financial relationships that could be construed as a potential conflict of interest.

## Publisher’s Note

All claims expressed in this article are solely those of the authors and do not necessarily represent those of their affiliated organizations, or those of the publisher, the editors and the reviewers. Any product that may be evaluated in this article, or claim that may be made by its manufacturer, is not guaranteed or endorsed by the publisher.
