# Supplementary material for: 18F-PSMA-1007 PET/CT Performance on Risk Stratification Discrimination and Distant Metastases Prediction in Newly Diagnosed Prostate Cancer
Source: Front Oncol. 2021 Oct 28;11:759053. doi: 10.3389/fonc.2021.759053 (PMC8581554; doi:10.3389/fonc.2021.759053)
Supplement: Supplementary file 1 [file Table_1.docx]

**Supplementary Table** Multivariate logistic analyses of factors predicting prostate cancer metastasis

| **Categorical variable** | Multivariate analysis | | |
| --- | --- | --- | --- |
|  | **OR** | **95% CI** | **P** |
| **SUVmax** | 1.081 | 1.003-1.165 | 0.040 |
| **Gleason Score**  **（high vs low-intermediate）** | 2.602 | 1.034-6.547 | 0.042 |
| **SUVmean** | 0.755 | 0.490-1.161 | P＞0.05 |
| **tPSA (high vs low)** | 1.466 | 0.579-3.708 | P＞0.05 |
